# Postoperative delirium in oral and maxillofacial surgery: a scoping review

**DOI:** 10.1186/s13005-024-00439-9

**Published:** 2024-07-23

**Authors:** Eman Alhammadi, Julian Max Kuhlmann, Majeed Rana, Helmut Frohnhofen, Henriette Louise Moellmann

**Affiliations:** 1 Cranio-and-Maxillo Facial Surgery, University Hospital Düsseldorf, Moorenstraße 5, Düsseldorf, 40225 Germany; 2 Heinrich-Heine-Universität Düsseldorf, Universitätsstrasse 1, Düsseldorf, 40225 Germany; 3 Orthopedics and Trauma Surgery, University Hospital Düsseldorf, Moorenstraße 5, Düsseldorf, 40225 Germany; 4 Dubai Health, Dubai, United Arab Emirates

**Keywords:** Postoperative delirium, Maxillofacial surgery, Risk management, Scoping review, Oral and maxillofacial surgery, Head and neck surgery

## Abstract

**Background:**

Postoperative delirium (POD) in the oral and maxillofacial settings has gained more attention in recent decades. Due to advances in medical technology, treatment possibilities have expanded treatment for elderly and frail patients. This scoping review explores the correlation between POD and oral and maxillofacial surgery, summarizing screening and management protocols and identifying risk factors in this surgical field.

**Methods:**

This review follows the Scoping Review extension of the Preferred Reporting Items for Systematic Reviews and Meta-Analyses (PRISMA-ScR). A comprehensive literature search was performed using multiple databases, focusing on articles published from 2002 to 2023 that discuss delirium in oral and maxillofacial surgery settings. The review was registered beforehand in the Open Science Framework (https://osf.io/r2ebc).

**Results:**

From the initial 644 articles, 68 met the inclusion criteria. These studies highlighted the significant heterogeneity in POD diagnosis methods. The review identifies multiple risk factors across the preoperative, intraoperative, and postoperative phases that influence the occurrence of POD. Significant and independent risk factors in multiple regression analysis were highlighted, creating a clinical prediction list for the occurrence of POD.

**Conclusion:**

It is crucial to preoperatively identify patients at risk for POD and actively modify these risks throughout the patient's hospital stay. Implementing nonpharmacological preventive measures for at-risk patients is recommended to decrease the incidence of POD. Future research should focus on creating standardized specialty-specific protocols incorporating validated assessment tools and addressing the full spectrum of risk factors associated with POD.

**Supplementary Information:**

The online version contains supplementary material available at 10.1186/s13005-024-00439-9.

## Background

The continuous advancement in medicine enables us to perform complex procedures on advanced-age patients. These patients are often frail and vulnerable due to multimorbidity, disease-related deconditioning, polypharmacy, and cognitive, functional, and social limitations. Preoperative consideration of these multiple factors is essential for care providers to achieve optimal results.

A common complication that develops in this population is postoperative delirium (POD). POD is considered a serious neuropsychiatric disorder that is associated with other underlying medical, cognitive, and functional impairments. However, it’s potentially preventable, reliably detectable, and can be effectively managed [1].

Several studies suggest that the incidence of POD in elective oral and maxillofacial surgery varies tremendously due to high heterogeneity in delirium diagnosis methods. An incidence rate between 3–37% has been reported [2–4]. That results in many short- and long-term adverse events and can impact a patient's life and have further financial consequences on the whole health system [5]. Regarding the severe consequences of POD, it seems plausible to identify patients at risk preoperatively, increase efforts for prevention and early detection.

POD is easier to prevent than to treat. Ludolph et al. reported that non-pharmacological multicomponent interventions could prevent almost a third of delirium cases in hospitalized patients [6]. It has been reported that 66% of patients with delirium are misdiagnosed [7]. Failure to diagnose delirium jeopardizes patients’ safety and impairs the treatment quality. Negative consequences following delirium, including emotional, functional, and cognitive distress, are documented widely in the literature [8]. A retrospective cohort study of more than 12,000 patients showed a 31% increased risk of dementia development following delirium. The risk of death five years postdiagnosis was even higher in patients over 65 years [9]. Delirium is also associated with post-traumatic stress disorder (PTSD). Grover et al. showed a 30% risk of developing PTSD following recovery from delirium. The severity of PTSD was also associated with delirium severity [10]. Common challenges in delirium diagnostics are the lack of awareness of the medical team, the variation of delirium presentation, its fluctuating nature, and hindered assessment in patients suffering from cognitive impairment. Clinicians tend not to follow a structured assessment for delirium or a score-based assessment but rather rely on general observational diagnosis. This practice could lead to an increased number of misdiagnosed patients [11–13].

Oral and maxillofacial surgery (OMFS) has a unique nature that carries multiple challenges, separating it from other surgical specialties. Patients' communication in the initial postoperative period may be limited. Therefore, relying on commonly used Delirium assessment tools might not always be feasible. Confusion Assessment Method (CAM) [14], Nursing Delirium Screening Scale (NuDESC) [15], and the (Alertness, Abbreviated Mental Test 4, Attention, Acute Chance or Fluctuating course) Test (4AT) [16] contain many items that depend on verbal communication. This might be impeded by intraoral and facial swelling, acute oral pain, limited mouth movement, and opening ability.

Additionally, tracheostomy might also complicate communication and detection of patients' incoherent and disorganized thinking in the initial period before speaking traning. Delirium symptoms like agitation may lead to patient refusal to any treatment, which further hinders delirium diagnosis [17]. Therefore, focusing solely on the postoperative phase in detecting and managing POD is not optimal clinical practice.

While several researchers have explored risk factors linked to POD following head and neck surgeries [18–21], there remains a notable gap in identifying the most suitable diagnostic methodology and optimal prehabilitation process.

This scoping review aims to explore the literature regarding the correlation between postoperative delirium and Oral and Maxillofacial Surgery. It aims to report the incidence of delirium among patients undergoing such procedures and the possibility of elevated risk in head and neck tumor patients. Furthermore, it identifies the assessment tools and methodologies frequently utilized for delirium detection in this surgical field, alongside pre- and postoperative management protocols. Additionally, it summarizes the OMFS-related risk factors reported in the literature.

## Methods

### Registration

This scoping review is conducted to systematically examine the available literature concerning postoperative delirium in the context of oral and maxillofacial surgery settings. The data is reported according to the Scoping Review extension of the Preferred Reporting Items for Systematic Review and Meta-Analysis statement (PRISMA-ScR) [22]. The protocol of this scoping review was prospectively registered on 22nd June 2023 in Open Science Framework (https://osf.io/r2ebc).

### Study design

#### Review questions

To identify important aspects related to delirium in patients after oral and maxillofacial surgical procedures, we developed research questions that clearly define the population, concept, and context (PCC) of this scoping review, following the recommendations of the Joanna Briggs Institute (JBI) [22] (Table 1).

**Table 1:** PCC framework

Participants (P)	Adults ≥ 18
Concept (C)	Postoperative Delirium
Context (C)	Oral and Maxillofacial surgery Head and Neck surgery

The goals of this review lie in analyzing the incidence of delirium in oral and maxillofacial surgery as well as short- and long-term outcomes of the patients. Moreover, which screening protocols are used, how risk factors are identified, and whether targeted preventive measurements may be applied to avoid POD.

#### Search strategy

Two independent authors performed a comprehensive search of the following databases: MEDLINE (via PubMed), Web of Science, Cochrane Library, Livivo, ScienceDirect, and Scopus, where the search strategy was tailored to each database. The search strategy included predefined keywords and subject headings related to delirium and OMFS. The detailed search strategy can be found in the supplemental files (Additional Table 5).

As main keywords, we used "Delirium", "Maxillofacial surgery" and "Head and neck surgery ". Furthermore, additional search terms were related to Delirium in oral and maxillofacial surgery: "Elderly", "geriatric", "cognitive impairment", "Alcohol Withdrawal Delirium", "Emergence delirium", "agitation".

All publications were analyzed by title and abstract to exclude irrelevant entries. Any discrepancy was resolved through discussion with a third researcher, and at the end of the selection process, full agreement was reached on the articles to be included. Full-text articles meeting the eligibility criteria were retrieved for further analysis. The database search included articles up until the 04.08.2023 Strict inclusion and exclusion criteria were applied. Articles were considered eligible if all inclusion criteria were met. Table 2 lists the inclusion and exclusion criteria.

**Table 2:** Inclusion and exclusion criteria

	Inclusion criteria	Exclusion criteria
Study design / Article type	Observational prospective and retrospective studies case series and case reports	letters to the editor Conferences Abstracts Book chapters
Period	All evidence published in the period 2002–2023	Publications prior to 2002
Language	English, German	Other languages
Additional criteria	NA	• Patient cohorts include a heterogeneous mix of different specialties, rendering the extraction of OMFS-related data not possible • No outcome was directly relevant to one of the primary research questions investigated in the review • Duplicate publication bias risk with systematic review and meta-analysis • Articles focused exclusively on emergence agitation

*NA* not applicable

The primary search results from the different databases were imported into Rayyan, a web-based program designed for study selection of systematic review and removal of duplication [23]. Title and abstract screening were done. Then, articles eligible for full-text screening were retrieved. In articles with mixed collection, head and neck surgery patients' results were extracted.

### Data extraction and charting

Extraction of information from the selected articles is known in scoping review as Data Charting. The authors reviewed the articles systematically and collected the information independently. The collected information was categorized into Bibliometric data, which contained the author’s name, publication year, and country(ies). Then, the study details include study design, population, setting, mean age, presence of cognitive impairment or psychological disorders, and surgical intervention. Additionally, details related to delirium were collected: Age of patients with delirium, delirium incidence, delirium assessment tool or diagnostic method, assessment frequency, management protocol, and short and long-term consequences related to delirium were extracted from all eligible studies. The data charting was carried out using Microsoft Excel. The article's details are summarized in a supplemental table (see Additional Table 1).

In articles examining risk factors for postoperative delirium, an additional charting process was conducted to distinguish between significant and non-significant risk factors in the preoperative, intraoperative, and postoperative periods. Additionally, independent risk factors were identified (see Additional Tables 2–4).

Since this is a scoping review, the quality of the included articles was not assessed. Narrative description and statistical analysis present a comprehensive overview of the findings. This is done following the Preferred Reporting Items for Systematic Reviews and Meta-Analyses extension for Scoping Reviews (PRISMA-ScR).

## Results

**Fig. 1:**
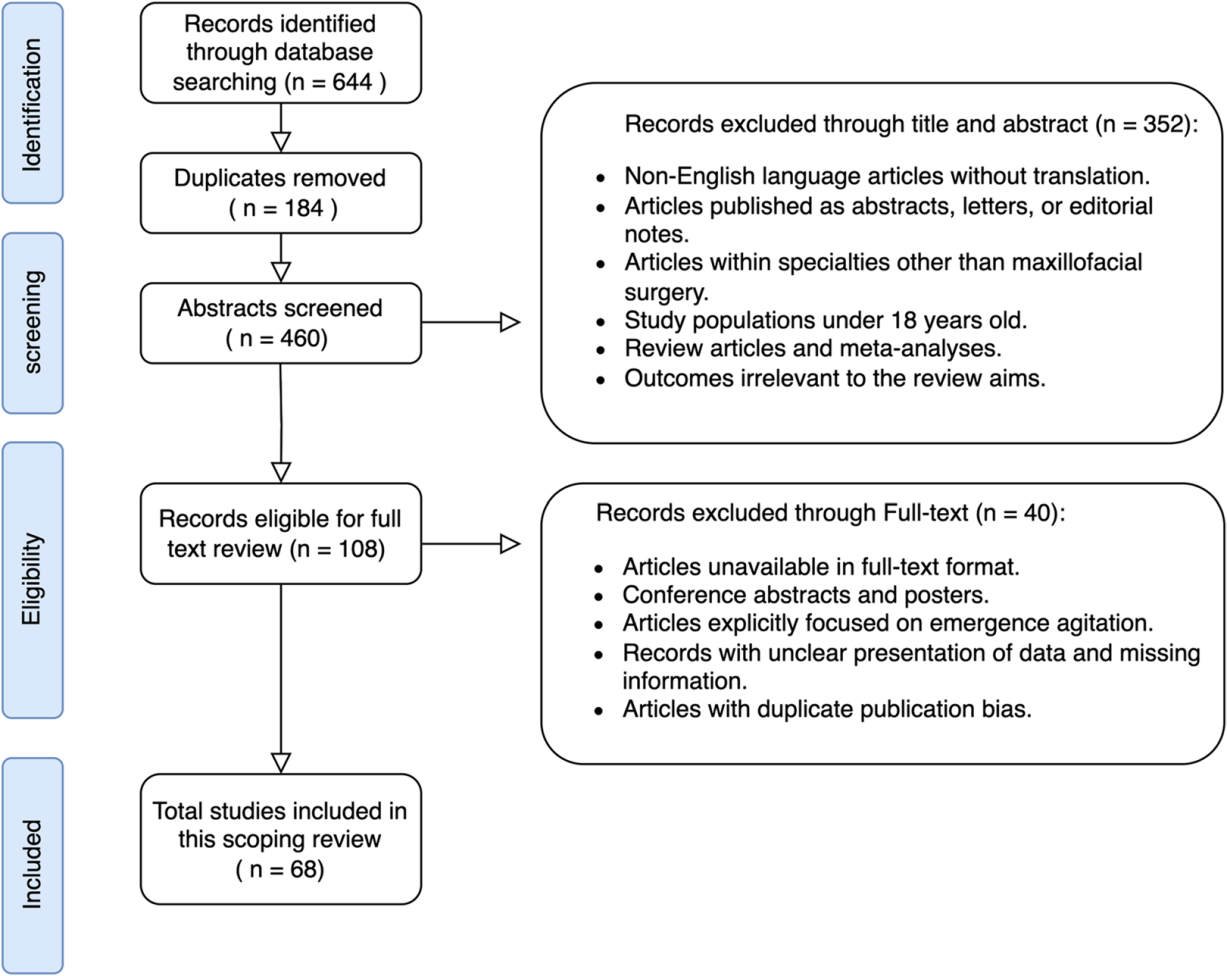
PRISMA chart for study selection

The literature search uncovered 644 relevant articles, with the process of identification, screening, and selection detailed in the Preferred Reporting Items for Systematic Reviews and Meta-Analyses (PRISMA) flow diagram (Fig. 1). At the title and abstract level, 108 citation records were deemed eligible for full-text review. Consequently, 68 studies met the eligibility criteria for final inclusion.

### Study characteristics

The characteristics of the included studies are individually summarized in Additional Table 1. Originating from diverse regions, 23.5% (*n* = 16) of the studies were from Japan [4, 19, 20, 24–36], 19.1% (*n* = 13) from the USA [5, 37–48], 14.7% (*n* = 10) from Germany and China [2, 21, 49–65], each of South Korea and the United Kingdom had (*n* = 3) studies 4.4% [66–71], and (*n* = 2) 2.9% from countries, including Australia [3, 72], Canada [73, 74], Taiwan [75, 76], and the Netherlands [77, 78]. One study included Brazil [79], India [80], Ireland [81], Israel [82], and Switzerland [83].

The included studies were highly heterogeneous in terms of designs and protocols followed. Retrospective cohorts and case–control studies design accounted for (*n* = 48) 70.6% [2–5, 19–21, 25–31, 33–39, 41–45, 49, 51–53, 55, 59–63, 66, 68, 70–72, 74–77, 81–83]. The studies with a primary focus on POD were mostly derived from retrospective data. Fifteen studies (22.1%) had either a prospective cohort design or a prospective cohort component designed as randomized or case–control studies [24, 40, 46–48, 50, 54, 57, 58, 64, 65, 67, 73, 78, 84]. Examining comparative aspects of interventions, risk factors, or other exposures. Two studies had mixed prospective and retrospective components [48, 69]. Additionally, (*n* = 3) 4.4% of case reports and series were included, where one reported delirium in patients operated in Local anesthesia and the other reported a young patient developing delirium after Orthognathic surgery [32, 79, 80].

Almost all the studies included in this scoping review took place in inpatient units and ICU settings. One study included POD assessments of patients in Post Anesthesia Care Unit (PACU) settings [2]. Also, one case report presented a patient developing delirium post-hospital discharge [79].

### POD-related information

Of the 68 primary studies in this scoping review, 14,662 subjects were examined, with 1417 (9.66%) diagnosed with postoperative Delirium. As most of the studies failed to report the type of POD, the question of the most occurring type in the OMFS field remained unanswered. The mean age of the patients who participated in the reviewed studies is 63.1 ± 10.6 years. The age of patients diagnosed with postoperative delirium was either unclear or unreported in *n* = 42 (61.8%) studies. The mean age reported of patients with delirium was 64.7 ± 11.3.

**Fig. 2:**
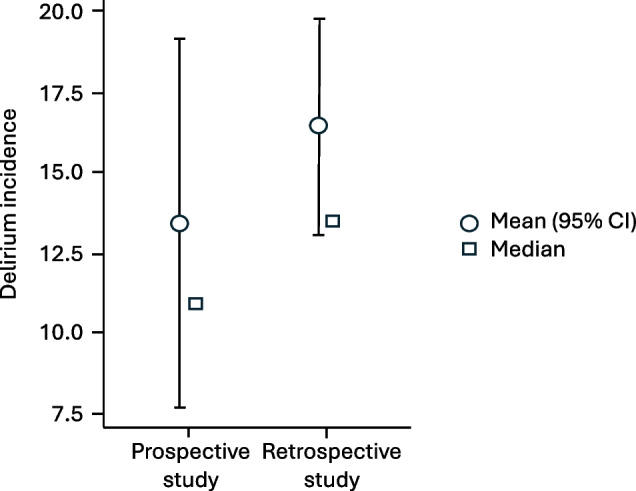
Comparison of POD incidence in prospective and retrospective studies

For comparison of the incidence of delirium in relation to the study design, 15 prospective and 48 retrospective studies were analyzed. The study population was 149 ± 180 patients in the prospective design and 129 ± 314 patients in the retrospective design. The incidence of delirium was 10.8 ± 11.3% and 13.4 ± 11.9% respectively. The comparison of the two groups showed no significant difference with U = 298; *p* = 0.325; *r* = 0.172. For the Prospective studies, the 25. – 75. Percentile was 4.21–21.8%; IQR 17.6%, while for the retrospective studies, it was 8.29–22.9%; IQR 14.7% (Fig. 2).

Examining the type of surgical interventions, 85.3% (*n* = 58) of the studies focused on individuals with head and neck cancer resection and reconstruction surgeries. In some articles (*n* = 6) 8.8% a mix of different oral and maxillofacial surgical procedures were presented [2, 33, 50, 56, 62, 83]. Two articles presented patients with Orthognathic surgery [37, 79] and one with condylar Fracture [80]. Only one article investigated cases of TMJ-related surgeries done under local anesthesia [32].

Baseline cognitive status was either unclear or unreported in (*n* = 50) 73.5% of the included studies. Similarly, preexisting psychiatric disorder was unreported in (*n* = 48) 70.6% of studies. Moreover, (*n* = 6) 8.8% of studies excluded patients with dementia, impaired cognitive status, or psychological disorders [21, 24, 25, 50, 56, 84].

The diagnostic criteria and/or validated assessment tools used to make the diagnosis of delirium varied. Numerous studies failed to report the assessment methods used to diagnose POD 50% (*n* = 34), particularly when it was not the primary focus of the research. In our analysis, we identified 9 distinct diagnostic methods used across these studies. Notably, in certain studies (*n* = 5), 7.4% solely relied on medical record notes with no reference to a diagnostic method or coding system [33, 35, 45, 73, 76].

The predominant tool observed across multiple studies was the The Diagnostic and Statistical Manual of Mental Disorders (DSM 3rd, 4th, 5th Edition) used in (*n* = 13) (19.1%) studies [4, 5, 19–21, 25, 28, 30, 34, 55, 66, 68, 71]. Confusion Assessment Method for the Intensive Care Unit (CAM-ICU) was applied in 5 (7.4%) studies [3, 21, 58, 65, 81]. CAM was also assessed in normal clinical setting in 2 studies [64, 84], and the 3D-CAM variation was applied in one study [50]. Other assessment methods used were Richmond Agitation-Sedation Scale (RASS) [53, 65, 83], Nu-DESC [2, 52], Intensive Care Delirium Screening Checklist (ICDSC) [24, 59, 83], Delirium Observation Scale (DOS) [83], and clinically manifest delirium based on the International Classification of Diseases, Tenth Revision (ICD-10) [2, 36, 55, 79]. It is noteworthy that additional psychiatric consultation was integrated in 6 (8.8%) studies [28, 30, 55, 66, 68, 71] and a Geriatricians consultation in one study [46].

Most of the studies failed to report their screening protocols, including the screening frequency and delirium resolution time (*n* = 58) 85.3%. A substantial degree of heterogeneity was evident among those studies that did outline a screening protocol. Screening protocols varied widely, ranging from single assessments in the Post-Anesthesia Care Unit (PACU) [2], to assessments every 4 h [24], every 8-h shift [59, 83], twice daily [52, 64, 65], or once daily [50, 58, 84]. Assessment duration ranged from 1 to 5 days. These discrepancies don’t adhere to the recommendations of the European Society of Anaesthesiology guidelines for postoperative delirium, which recommend initiating screening in the PACU and continuing throughout each shift until postoperative day 5, utilizing a validated scoring tool [85]. This inconsistency reveals a considerable misalignment between research practices and established clinical guidelines and directly affects the reported delirium incidence rates. This impacts the early detection and the implementation of timely management strategies in clinical settings.

In the studies reviewed, 76.5% (*n* = 52) included no specific protocols for managing postoperative delirium. Nonpharmacological approaches were reported in (*n* = 5) 7.4% studies [59, 65, 68, 79, 83], while others relied on a combined approach or purely pharmacological management strategies (*n* = 15) 22.1% [19–21, 25, 30, 34, 48, 50, 58, 68, 76, 79, 80, 83]. Administered medications included antipsychotics (haloperidol, risperidone, quetiapine, pipamperone), antidepressants ( escitalopram, mirtazapine), anxiolytics, and sedatives (diazepam, dexmedetomidine, clonidine) and pain medication as PCA-fentanyl as part of the treatment in different doses and frequencies. Assessing the effectiveness of these management protocols is challenging due to the considerable heterogeneity of delirium diagnostic tools and timing. Recent guidelines advocate for initially employing nonpharmacological strategies as the primary management method. Should pharmacological treatments be necessary, they must be administered by minimizing dosage and duration [86, 87]. Clear preventive measures protocols were not reported.

Some studies have documented adverse outcomes and long-term consequences linked to POD. Patients experiencing post-operative delirium showed a significantly higher rate of postoperative complications [21]. These include increased incidences of postoperative wound complications and pulmonary complications [70], extended durations of hospitalization [55, 73], and an increased necessity of discharge to post-care facilities [38]. Additionally, prolonged mechanical ventilation and an elevated risk of requiring unplanned secondary tracheotomy [49]. Further findings indicate delayed mobilization post-surgery [28, 74] and a decreased overall survival rates [60].

### POD risk factors

In this scoping review, 28 included studies examined various hypothesized risk factors in the preoperative, intraoperative, and postoperative phases (Additional Tables 2–4). The factors that showed a statistically significant relation to delirium in at least one of the studies were mentioned. Factors without information regarding their significance were either not assessed, or no specific association was mentioned in the research findings.

The Significant association was marked as (√), Factors with a significant association that were identified as Independent factors in multiple regression analysis were marked as (√*), and nonsignificant factors were marked as (x).

#### Preoperative risk factors

##### Patient’s characteristics

The most distinct risk factor is increased age, of the 23 studies investigated age as risk factor 16 studies reported a significant association (*n* = 16/23) 69,6% [3, 5, 19, 20, 30, 31, 33, 34, 47, 55, 60, 68, 70, 71, 75, 88], and eight of them reported it as an independent risk factor. Age is accompanied by increased comorbidity and decreased regenerative capability and has often been described as a risk factor in other surgical specialties [55].

The data on the male gender in regard to POD remains inconclusive. In four studies (*n* = 4/20) 20% reported it as a significant risk factor [5, 30, 34, 52], while sixteen failed to find a significant association.

Concerning increased BMI, no study found a significant association with POD. On the other hand, two studies found a significant association between history of malnutrition and POD [4, 5], and one described a low skeletal muscle index as an independent risk factor for hypoactive delirium [4].

##### Laboratory data

Contradictory, both high [4], and low [20, 52, 68] preoperative albumin have been found as significant risk factors (*n* = 4/8) 50% [4, 20, 52, 68]. Low albumin levels indicate poor nutrition or metabolic disturbances. Researchers suggest that high albumin levels do not lead to POD per se. High Albumin levels might allude to dehydration. Also, a steep perioperative drop in Albumin levels indicates malnutrition or inflammation, which could promote POD. Maintaining sufficient hydration and albumin levels could be beneficial [4]. Counterintuitively, a preoperative high hemoglobin was found as a significant risk factor by two studies (*n* = 2/8) 25% [21, 70]. This could also indicate dehydration, which leads to increased vulnerability for POD [70]. Low TSH was also found to be a significant risk factor [55].

##### Pre-existing medical condition

Pre-existing medical conditions are routinely assessed and deliver ample information about the patient’s vulnerability. Firstly, an American Society of Anesthesiologists score (ASA) of III/IV was displayed as both significant (*n* = 5) [3, 19, 47, 60, 68] and independent (*n* = 2) [60, 68] risk factor (*n* = 5/9) 55,5%, indicating severe health condition. Furthermore, recent hospitalization [30] and a history of delirium [2] are recognized as significant risk factors.

A special focus lies on neurological disorders. Cognitive impairment and dementia were found as independent risk factors (*n* = 4/7) 57,1% [2, 33, 47, 71], indicating reduced cognitive reserves.

In addition, psychiatric disorders (*n* = 2/9) 22,2% [3, 68] and especially alcohol abuse (*n* = 5/18) 27,8% [5, 21, 42, 47, 70] proved to be significant risk factors. Alcoholism is a peculiarity of OMFS patients, as alcohol and tobacco are one of the main risk factors for head and neck cancer. Researchers used alcohol-drinking questions in their ‘clinical prediction rule’ for POD [47]. Vice versa, preoperative abstinence showed a protective effect [5]. Identifying alcohol misuse and establishing perioperative abstinence constitutes a beneficial target.

Less apparent, smoking was also a significant risk factor in three studies (*n* = 3/15) 20% [4, 5, 70]. Negative effects on the cardiovascular system are widely reported. This might impede the perfusion of the brain and reduce neuro-cognitive reserves. Thereby, the vulnerability for POD could be increased.

Other conditions significantly associated with POD include diabetes mellitus (*n* = 4/9) 44,4% [4, 5, 30, 55], hypertension (*n* = 2/6) 33,3% [19, 84], cardiovascular disease (*n* = 2/8) 25% [5, 70], renal dysfunction (*n* = 2/5) 40% [5, 19], COPD (*n* = 2/3) 66,6% [30, 70], tumor stage IV (*n* = 2/8) 25% [35, 89], and sleep disorders (*n* = 1/2) 50% [68]. No association was found between a history of chemo-/ radiotherapy and POD.

##### Medication

A significant association was found between the use of insulin/antidiabetics [55], psychotropic medication [4], and irregular antihypertensive medication use [84]. The associations might not be causal but due to underlying diseases. Nonetheless, a preoperative check and adjustment should be considered, focusing on psychotropic and antihypertensive medication.

#### Intraoperative precipitating factors

The most important intraoperative risk factors are duration of surgery (*n* = 11/23) 47,8% [19, 20, 28, 35, 45–47, 52, 70, 88, 89] and duration of anesthesia (*n* = 2/4) 50% [28, 33], as they go along with increased stress, tissue damage and blood loss. Accordingly, intraoperative blood loss (*n* = 3/7) 42,9% [28, 46, 52], low hemoglobin (*n* = 1/2) 50% [20], elevated blood lactate [21], blood transfusion (*n* = 5/9) 55,6% [19, 20, 35, 70, 89], and crystalloid infusion (*n* = 3/8) 37,5% [35, 52, 89] were identified as significant risk factors, indicating a lack of oxygenation. Moreover, surgical reconstruction (*n* = 6/13) 46,1% [21, 28, 34, 35, 45, 55] and tracheotomy (*n* = 1/11) 9,1% [68] are described as significant risk factors. This might allude to the severity of the surgery. On the other hand, neck dissection was not found to be a significant risk factor. Therefore, surgery should be kept as short and non-invasive as possible. Blood loss should be kept to a minimum. Hemoglobin and blood pressure should be stabilized if possible.

#### Postoperative risk factors

Certain precipitating postoperative factors for patients at risk result from a severe clinical course, which itself is a risk factor for POD. These are ICU duration (*n* = 7/10) 70% [2, 20, 21, 30, 53, 55, 68], hospitalization duration (*n* = 5/10) 50% [20, 21, 55, 70, 71], ventilation duration (*n* = 2/4) 50% [30, 52], time to mobilization (*n* = 2/5) 40% [28, 71], impaired wound healing (*n* = 1/5) 20% [70], transplant revision (*n* = 1/5) 20% [21] and pulmonary complications (*n* = 1/2) 50% [70]. The potential to optimize these risk factors is limited. However, early mobilization and discharge from the ICU should be pursued if feasible.

Postoperative laboratory risk factors correlate with intraoperative risk factors: Again, low hemoglobin (*n* = 4/8) 50% [20, 28, 30, 34], low hematocrit (*n* = 2/5) 40% [28, 34], low red blood cell count [28, 34], and increased blood lactate (*n* = 1/2) 50% [21] all indicate a lack of oxygenation. Maintaining a stable hemoglobin level perioperatively, and therefore ensuring brain oxygenation, seems beneficial for POD prevention. Further risk factors are increased IL-6 [88], indicating inflammation, and increased potassium levels (*n* = 1/6) 16,7% [30], indicating cellular damage or metabolic disturbances.

The findings on postoperative medication are contradictory. The application of major tranquilizers [30] and psychotropics [55] were found to be risk factors for POD. Conversely, a lack of minor tranquilizers [35] was shown to be an independent risk factor. The causality remains unclear as tranquilizers/ psychotropics may have been applied to treat POD. It is suggested that minor tranquilizers stabilize the sleep–wake cycle and potentially prevent POD [35, 90]. Accordingly, one study highlighted postoperative insomnia as an independent risk factor [4]. Findings from both pre-and postoperative periods indicate a correlation between medications having anticholinergic effects and POD. Thus, preoperative evaluation of a patient's anticholinergic cognitive burden using a straightforward score, like the Anticholinergic Cognitive Burden (ACB) score, could be beneficial [91].

Furthermore, one study found patient-controlled analgesia (PCA) via fentanyl superior to morphine [34]. In comparison, the application of morphine was significantly associated with POD. This effect may result from better pain control via fentanyl and less impact on the sleep–wake cycle [34].

Another risk factor was the application of a new medication postoperatively [55].

Additionally, in the OMFS setting, nutrition plays a crucial role. The patient’s ability of oral food intake is often compromised. An increased Nutritional Risk Screening (NRS) score [53] was found as an independent risk factor. Coherently, a perioperative drop in total protein (*n* = 1/2) 50% [34], low postoperative albumin (*n* = 1/3) 33,3% [70], and performing percutaneous endoscopic gastrostomy postoperatively [55] posed risk factors. These factors suggest nutritional deficiencies that might contribute to the emergence of POD. Ensuring sufficient nutrition throughout the perioperative period should be aimed for.

## Discussion

In literature, the terminology related to delirium in the context of oral and maxillofacial research is often used interchangeably and inconsistently. Terms such as "delirium," "postoperative delirium," "acute cognitive impairment," "emergence delirium," and "delirium tremens" are sometimes used without clear clarification of the definition. This leads to confusion among readers and researchers. Sometimes, these terms are used with a broad, encompassing meaning, hinting at their association with postoperative delirium rather than representing distinct and narrow categories as the term implies. The lack of standardized language usage creates additional challenge in understanding and comparing findings across studies. This emphasizes the need for unified terminology and precise definitions to establish a mutual understanding of this complication [92].

### Incidence variability

The incidence of delirium in oral and maxillofacial studies exhibits significant variability due to numerous factors, including the inhomogeneity in study designs, different data collection methods, participant selection criteria, and the classification of outcome measuring protocols.

Although we didn’t find a significant difference in the reported incidences between prospective and retrospective studies, methodological heterogeneity remains an issue across studies. Most studies followed a retrospective methodology that lacks clear and standardized assessment methods. This leads to inconsistencies in identifying delirium cases. Collecting the information only from the patient's medical records with no previous guidelines of how delirium is regularly assessed postoperatively can lead to an overestimation of delirium or a lack of documentation of hypoactive cases. This approach results in incomplete and fragmented datasets, as delirium is mentioned as a side note or incidental finding. The absence of scoring assessment systems or structured assessments can skew the results, accounting for misleading conclusions.

Another drawback of retrospective studies is the lack of comprehensive real-time assessments, which is crucial given delirium's transient and fluctuant nature.

Furthermore, delirium is often not the primary focus of these studies; instead, it is considered one of the many complications that may arise postoperatively. This also can lead to underreporting or insufficient attention to delirium cases during the research period. Additionally, using different assessment methods further complicates the presented data.

### OMFS patients in the postoperative period

Communication with patients following oral and maxillofacial surgery presents a unique challenge, necessitating the development of a specialized methodology for assessing postoperative delirium in this patient population. This distinctiveness arises from several factors related to OMFS procedures, including patients’ intraoral and facial swelling, acute oral pain, facial flaps, speech impairment due to edema, and limited mouth opening. Not rarely are these factors combined with the presence of tracheostomy and nasogastric tube interventions. These factors collectively hinder the conventional approach to delirium assessment. They heavily rely on verbal communication to assess the patient's orientation, attention, disorganized thinking, and communicative competence.

Increasing preoperative patient awareness about delirium, its significance, and how it will be assessed postoperatively is crucial. Providing them with alternative response methods to the assessment questions, such as writing or utilizing visual aids, could facilitate delirium evaluation. This can encourage patients to participate in their assessment despite speech limitations or discomfort. These unique postoperative circumstances may necessitate developing a new assessment tool or modifying conventional assessment tools.

Another aspect concerning POD in OMFS patients is that POD and its complications can delay the initiation of necessary adjuvant chemoradiotherapy for head and neck carcinoma, where an early start of treatment is essential for successful cancer management [30].

### Delirium underdiagnosed in OMFS

The underdiagnosis of delirium in oral and maxillofacial studies primarily originates from the absence of routine screening protocols. Delirium is often mentioned as a secondary finding, among many complications, rather than a primary research focus. The lack of active and systematic screening in these studies particularly leads to the underdiagnosis of the hypoactive form of delirium. Due to its nature, this is challenging to identify without proactive measures.

Another contributing factor to underdiagnosis is the lack of awareness among surgeons and the supporting medical teams regarding delirium screening and management methodologies [93]. The impact on a patient's physical and psychological well-being and the strain on family members is often overlooked [94]. The patient is usually perceived as ‘the sleepy, quiet patient’ or ‘the uneasy, restless patient’. To improve this part of clinical practice, proactive screening is needed by implementing routine screening protocols and utilizing validated assessment tools. Also, it needs to be recognized that delirium presents itself in diverse clinical forms, including hypoactive form.

The absence of a standardized diagnostic approach and diagnostic code poses a challenge. It results in the oversight of documented episodes of delirium in a patient's medical history. It is reported that a history of delirium increases the risk of its recurrence and can be a predictor of cognitive impairment and dementia [95]. Therefore, it is crucial to ensure the inclusion of such information in medical records using standardized coding systems like the ICD code.

### Preoperative cognitive assessment

The absence of preoperative cognitive assessments in routine practice presents another challenge in diagnosing postoperative delirium. Without a baseline cognitive reference point, identifying deviations becomes notably difficult. Some included studies in this scoping review excluded patients with cognitive impairments to eliminate this predisposing factor. Therefore, establishing a comparative baseline through efficient, validated cognitive tests such as the clock drawing test, six-item screening test, and months backward test can provide preoperative insights into patients' cognitive function. In the DELPHIC study, superior baseline cognitive function was associated with a reduced risk of delirium (RR: 0.63, 95% CI: 0.45–0.89), and when delirium occurred, it was generally less severe and of shorter duration [96]. By integrating simple, time-efficient assessments into preoperative protocols, healthcare practitioners can proactively identify at-risk patients, paving the way for more targeted interventions and improved postoperative outcomes.

### Risk factors

As medical advances enable the treatment of increasingly complex cases in older or more frail patients, identifying their OMFS-specific risk factors becomes crucial for delirium prevention. Several validated tools, such as DRAS [97], DRAT [98], and DEAR [99], exist for assessing delirium risk. These are simple, time-efficient bedside tests that don’t require special training and combine the main general risk factors for POD. However, none of the published studies in this scoping review utilized these tools to evaluate at-risk patients. Patients who could be screened routinely to detect high-risk individuals before surgery. Subsequently, the necessity of surgery may be evaluated. Pre-, intra-, and post-operative risk factors could be optimized, and further preventative measures could be implemented. Our scoping review focused on identifying independent risk factors in multiple regression analysis for POD; we found numerous predictors across the preoperative, intraoperative, and postoperative periods. The predictors in the preoperative period included increased age, male gender, higher ASA classification (III, IV), the presence of psychiatric disorders, cognitive impairments including dementia, alcohol abuse, smoking, hypertension, low skeletal muscle mass, albumin levels, and irregular use of hypertension medication. Intraoperatively, factors such as surgery duration, high fluid intake, surgical reconstruction using free fibular grafts, and low hemoglobin levels were identified as independent risk factors. Postoperatively, the demanding environment following reconstructive surgery with vascular anastomosis in the head and neck region can significantly stress patients, potentially triggering delirium. The predictors in the postoperative periods were elevated serum IL-6 levels, prolonged ventilation, delayed mobilization, extended hospital stay, insomnia, pulmonary complications, the necessity for transplant revision, and nutritional risk.

## Conclusions

In conclusion, it's essential to identify patients at risk of POD preoperatively and adjust the risk factors whenever possible from admission to discharge. Implementing targeted nonpharmacological prophylactic strategies for those at high risk can help reduce POD and enhance prompt intervention. Future research should investigate adjustable risk factors, particularly for elective surgical procedures, and develop standardized, specialty-specific protocols that include validated assessment tools. Additionally, training programs for healthcare professionals and educational initiatives for families and caregivers are vital, alongside policies and guidelines, to ensure these practices are effectively integrated across healthcare settings. All of this will provide a deeper understanding of delirium in the OMFS setting, its common subtypes, prognoses, and therapeutic options, bridging existing knowledge and practice gaps.

### Supplementary Information

Supplementary Material 1.

Supplementary Material 2.

Supplementary Material 3.

Supplementary Material 4.

Supplementary Material 5.

## Data Availability

Data is provided within the manuscript or supplementary information files.

## Abbreviations

4AT: Alertness, Abbreviated Mental Test 4, Attention, Acute Chance or Fluctuating course
ACB: Anticholinergic cognitive burden
ASA: American Society of Anesthesiologists
BMI: Body-mass-index
CAM: Confusion Assessment Method
CAM-ICU: Confusion Assessment Method for the Intensive Care Unit
COPD: Chronic obstructive pulmonary disease
DELPHIC-study: Delirium and Population Health Informatics Cohort study
DEAR: Delirium Elderly At-Ris
DOS: Delirium Observation Scale
DRAS: Delirium Risk Assessment Score
DRAT: Delirium Risk Assessment Tool
DSM: Diagnostic and Statistical Manual of Mental Disorders
Hb: Haemoglobin
ICD-10: International Classification of Diseases, Tenth Revision
ICDSC: Intensive Care Delirium Screening Checklist
ICU: Intensive Care Unit
IL-6: Interleukine 6
NA: Not applicable
NR: Not reported
NuDESC: Nursing Delirium Screening Scale
OMFS: Oral and maxillofacial surgery
PACU: Post-Anesthesia Care Unit
PCA: Patient-controlled Analgesia
PCC: Population, Concept, and Context
POD: Postoperative delirium
PRISMA-ScR: Preferred Reporting Items for Systematic Reviews and Meta-Analyses extension for Scoping Reviews
PTSD: Post-traumatic stress disorder
RASS: Richmond Agitation-Sedation Scale
TMJ: Temporomandibular joint
TSH: Thyroid Stimulating Hormone
